# Etiopathogenesis and Emerging Methods for Treatment of Vitiligo

**DOI:** 10.3390/ijms24119749

**Published:** 2023-06-05

**Authors:** Tomasz Iwanowski, Karol Kołkowski, Roman Janusz Nowicki, Małgorzata Sokołowska-Wojdyło

**Affiliations:** 1 Dermedica, Aleja Zwycięstwa 34, 80-219 Gdansk, Poland; tomasziwan@gmail.com; 2 Dermatological Students Scientific Association, Department of Dermatology, Venerology and Allergology, Faculty of Medicine, Medical University of Gdansk, 80-214 Gdansk, Poland; karolkolkowski@gumed.edu.pl; 3 Department of Dermatology, Venerology and Allergology, Faculty of Medicine, Medical University of Gdansk, 80-214 Gdansk, Poland

**Keywords:** vitiligo, pathogenesis, treatment, Janus kinase inhibitors

## Abstract

Vitiligo is an acquired chronic depigmenting disorder of skin. It is mostly asymptomatic and characterized by amelanotic macules and patches that affects 0.5% to 2% of the world’s population. The etiology of vitiligo has not been clearly elucidated and multiple theories have been proposed regarding the causes of the disorder. Among the most prevalent theories, the genetic predisposition, oxidative stress theory, promotion of cellular stress and pathologic influence of lymphocytes T have been highlighted. As a result of increases in in-depth knowledge concerning the pathogenetic processes in vitiligo, we review the most recent information concerning its etiopathogenesis and treatment methods including topical and oral Janus kinase inhibitors, prostaglandins and their analogues, namely afamelanotide, Wnt/β-catenin-signaling agonists and cell-based therapies. Topical ruxolitinib has been registered for vitiligo treatment, whereas other agents as oral ritlecitinib, afamelanotide and latanoprost have been studied in ongoing clinical trials. New highly effective therapeutic strategies may be developed thanks to molecular and genetic studies.

## 1. Introduction

Vitiligo is characterized by patchy skin depigmentation that can be present on any part of the body. Approximately 1% of the world’s population is affected without any significant difference in prevalence due to sex, ethnicity or geographic region [1]. Vitiligo negatively influences patients’ quality of life by decreasing self-esteem and causing significant psychological distress [2,3,4]. The etiology of vitiligo has not been clearly elucidated. Multiple theories have been proposed regarding the causes of the disorder: genetic, oxidative stress (autotoxicity), autoimmune, autoinflammatory, neurogenic, melanocyte detachment (melanocytorrhagy), apoptotic and multifactorial [5]. While the detailed molecular mechanisms still require further investigation, recent studies have revealed that interferon gamma (IFN-γ) and the chemokine ligand 9/10-chemokine receptor 3 (CXCL9/10-CXCR3) axis appear important in vitiligo via inhibiting melanogenesis, inducing apoptosis of melanocytes and further recruiting T cells to the skin [6]. They all have been involved in the Janus kinase/signal transducer and activator of transcription (JAK/STAT) pathway. In addition, cytokines, including IL-15, IL17/23 and TNF α as well as the Wnt/β-catenin signaling pathway, Tregs, HSP70i and several miRNAs have also been proven to be involved in the pathogenesis of vitiligo. Therefore, our aim was to discuss recent knowledge concerning the pathogenesis of vitiligo and analyze emerging treatment methods of that disease.

## 2. Etiopathogenesis

### 2.1. Genetics of Vitiligo

Both genetic and environmental components have been involved in the pathogenesis of vitiligo. From the genetic point of view, vitiligo behaves as a typical polygenic condition with a small contribution of individual specific genetic factors. There is no family history in more than 90% of cases. There is more than one close relative affected in 9% of cases. The relative risk of vitiligo for the first-degree relatives is increased 7–10-folds. Early segregation analyses of such families had suggested additive, polygenic inheritance with heritability between 50% and 75% [7,8]. A polygenic nature of vitiligo is also supported by results of subsequent genome-wide association studies (GWASs) that have identified 50 contributory loci and each locus contributes just slightly to overall vitiligo heritability [9,10,11]. GWAS has been established as the “gold standard” in detecting predisposition genes of vitiligo development. Approximately 90% of the “suspicious” genes encode immunoregulatory proteins, while the remaining 10% encode melanocyte proteins. The identified vitiligo-associated genes are critical for antigen presentation (e.g., MHC class I and class II), T-cell development (e.g., CD44 and SH2B3), T-cell receptor signaling (e.g., PTPN22 and UBASH3A), T-cell activation (e.g., CTLA4, IKZF4 and CD80), melanocyte homeostasis and melanogenesis (e.g., TYR, OCA2, MC1R and ASIP) and apoptosis (e.g., RERE, GZMB, CASP7, FASLG, BCL2L11, BCL2L12, SERPINB9, NEK6 and BAD) [9]. Similarly to other genetically complex diseases, most vitiligo GWAS loci involve common, low-to-moderate effect-size variants. Moreover, similarly to in other autoimmune diseases, the corresponding identified genes involve the regulation of immune cells and apoptosis, as well as melanocyte components that can act as autoantigens. Multiplex-vitiligo-affected families segregate the high burden of the common, low-effect risk alleles identified by GWAS. Furthermore, the risk score is roughly proportional to the number of affected relatives within a family, suggesting that high polygenic risk is an important contributor to overall genetic risk for vitiligo in both smaller and larger multiplex-affected families.

### 2.2. Oxidative Stress Leading to Autoimmunity

Oxidative stress may be the initial event in melanocyte destruction. Indeed, melanocytes from vitiligo patients were noted to proliferate more slowly than those in healthy controls [12]. Moreover, they also demonstrated dysregulated redox balance associated with low expression of catalase [13]. Catalase protects cells from reactive oxygen species (ROS) by reducing hydrogen peroxide to oxygen and water, and melanocytes in particular produce high levels of ROS as a by-product of melanin production. Xenobiotic phase I (cytochrome P450s) and phase II enzymes (i.e., glutathione S-transferases [GSTs] and N-acetyltransferases [NAT1 and NAT2]) play a major role in the biotransformation and protection against environmentally exposed exogenous and endogenous toxins, i.e., drugs, carcinogens, hair dyes and several others [14,15,16]. N-acetyltransferase metabolizes a wide range of xenobiotic compounds that may be responsible for ROS production and melanocyte damage via the acetylation process [14,17]. It catalyzes metabolic inactivation or activation of environmentally exposed compounds, such as the plethora of hydrazine and arylamines by the pathways of n- or o-acetylation [17]. Dysregulated redox balance was reported within vitiligo patient skin, which had elevated hydrogen peroxide levels and increased oxidative by-products [13,18]. This causes widespread alteration of the antioxidant system: an imbalance of elevated oxidative stress markers (superoxide dismutase, malondialdehyde and ROS) and a significant depletion of antioxidative mechanisms (catalase, glutathione peroxidase, glutathione reductase, thioredoxin reductase and thioredoxin, superoxide dismutases and the repair enzymes methionine sulfoxide reductases A and B) in the skin and in the blood [19]. An absence of activity and deregulation of acetylcholinesterase (AchE) due to H2O2-mediated oxidation further maintains epidermal oxidative stress. H2O2-induced oxidative injury increases ATP release from keratinocytes. The high concentration of extracellular ATP is inducing both ROS production and cell death in melanocytes. Oxidative stress promotes the secretion of CXCL16 that induces CXCR6+CD8+ T cell migration to the skin [14,17,18,20].

### 2.3. Damage-Associated Molecular Patterns (DAMPs)

Cellular stress may push melanocytes to secrete exosomes which contain melanocyte-specific antigens, miRNAs, heat shock proteins and damage-associated molecular patterns (DAMPs). DAMPs activate dendritic cells to produce proinflammatory cytokines [21]. The heat shock protein HSP70i is known as the main DAMP involved in the pathogenesis of this disease. By binding to the melanocyte-specific melanosomal proteins/peptides, it assists in protein folding and transport and potentially MHCI/II loading. HSP70i exposure boosts dendritic cell activation by processing and presenting antigens measured by the expression of dendritic cell maturation markers such as CD80 and CD83 [22]. Chemically induced cellular stress also intensifies the synthesis of the receptor NLRP3 participating in the activation of the inflammasome or the cytokine IL-1β directly. This is followed by cytokine- and chemokine-driven activation of T helper 17 cells and the dysfunction of T regulatory cells. Overexpression of the gene encoding the receptor NLRP1 (Langerhans cells) leads to the activation of inflammasome and induces the conversion of pro-IL-1β into active IL-1β, which is involved in the pathogenesis and progression of vitiligo [23].

### 2.4. The Role of CD8+ T-Cells

The activation of adverse exogenous and/or endogenous factors in genetically predisposed individuals induces cellular stress in melanocytes and stimulates autoimmune and autoinflammatory processes. High numbers of cytotoxic CD8+ T cells have been identified in the blood and perilesional skin of vitiligo patients and these cells exhibit antimelanocyte cytotoxic reactivity. They produce a pore-forming protein, perforin, that forms transient pores on the surface of target cells and provides a short window of time for the direct entry of granzymes into the cytosol. The granzymes induce apoptosis through multiple pathways inside the cell [24]. Autoreactive cytotoxic CD8+ T cells not only engage melanocyte destruction but also promote disease progression through the local production of IFN-γ and TNF-α. IFN-γ-induced chemokines are then secreted from surrounding keratinocytes and activate the JAK-STAT signaling pathway, leading to the release of the chemokines CXCL9 and CXCL10. They promote migration of further autoreactive T cells (CD8 +) by attaching to their receptor (CXCR3), which exacerbates inflammation via the positive feedback mechanism. IFN-γ inhibits melanogenesis and directly induces melanocyte apoptosis [25]. Therapies that disrupt the pathway targeting IFN-γ, the IFN-γ receptor, the downstream signal JAK-STAT pathway, CXCL10 and its receptor CXCR3 are the most promising in vitiligo management.

### 2.5. The Role of Trm Cells

**Figure 1:**
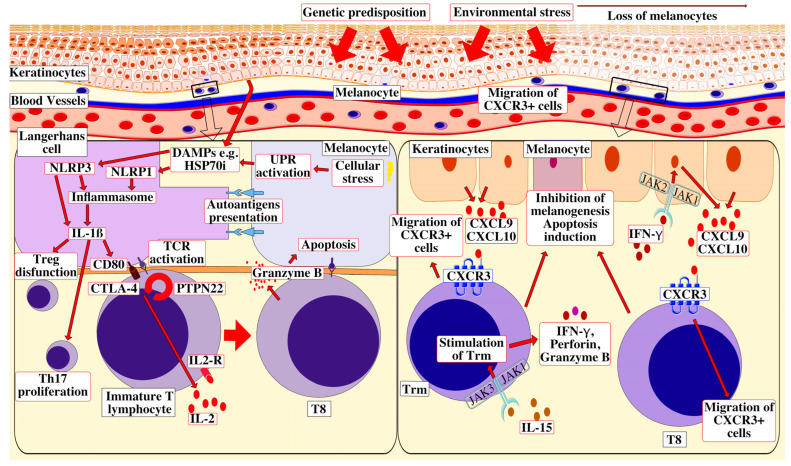
Vitiligo pathogenesis and genetics. Skin is damaged by UV or other trauma. Molecules displaying DAMPs interact with NLRP1 in the cytoplasm of Langerhans cells, stimulating nucleation of an NLRP1 inflammasome, thereby activating caspases that cleave the IL-1β precursor to biologically active secreted IL-1β. Chemically induced cellular stress also intensifies the synthesis of the receptor NLRP3 participating in the activation of the inflammasome or the cytokine IL-1β directly. Next, IL-1β activates T helper 17 cells and the dysfunction of T regulatory cells. Langerhans cells take up peptide autoantigens presented by HLA class I molecules expressed on the surface of nearby melanocytes, including peptides derived from TYR, OCA2 and MC1R, and these peptide autoantigens are then transferred to HLA class II molecules expressed on the Langerhans cells’ surface. Stimulated by IL-1β and facilitated by the interaction of CD80 with CTLA4 and by the action of PTPN22, these melanocyte-derived peptide autoantigens are then presented to immature T cells that express cognate TCR, the response of which is regulated by PTPN22. The activated T cells express IL-2, which binds to the IL-2 receptor expressed on their surface, stimulating maturation to T8 cells that express GZMB. The TCR expressed by these autoreactive T8 cells binds its cognate autoantigen presented on the surface of target melanocytes by HLA class I molecules and GZMB is introduced into the target melanocyte, inducing apoptosis. On the right hand, T8 expression of IFN-γ in vitiligo lesions activates the JAK/STAT pathway after binding to the IFN-γ receptor, thus facilitating the release of CXCL9/10 from the keratinocytes. The binding of CXCL9/10 to CXCR3 increases CXCR3+ T cell recruitment and, along with the depigmentation, more T-cells appear at the site of inflammation. The pathogenic function of IL-15-dependent Trm is to produce IFN-γ, perforin and granzyme B: cytokines with cytotoxic properties. Along with T8, the Trm cells contribute to inhibition of melanogenesis and induce the apoptosis of melanocytes. Abbreviations: UV—ultraviolet, DAMPs—damage-associated molecular patterns, NLRP1—NOD-like receptor thermal protein domain associated protein 1, IL—interleukin, NLRP3—NOD-like receptor thermal protein domain associated protein 3, HLA—human leukocyte antigen, TYR—tyrosinase, OCA2—melanosomal transmembrane protein, MC1R—melanocortin-1 receptor, CTLA4—cytotoxic T cell antigen 4, PTPN22—protein tyrosine phosphatase non-receptor type 22, TCR—T-cell receptors, GZMB—granzyme B, T8—cytotoxic CD8+ lymphocyte T, Trm—resident memory T cells; JAK—Janus kinase, STAT—signal transducer and activator of transcription, CXC9/10—chemokine (C-X-C motif) ligand 9/10, CXCR3—chemokine (C-X-C motif) receptor 3 and IFN-γ—interferon-gamma.

Relapse of vitiligo is often observed during the first year after stopping treatment and occurs mainly at the same areas that were previously involved. This phenomenon suggests a role of autoimmune memory within the inflammatory site, namely resident memory T (Trm) cells. They are a long-lived subset of T cells that remain within most nonlymphoid tissues following T cell-driven inflammation and are marked by the expression of surface markers CD69, CD103 and CD49a. After stimulation, they also express CXCR3, IFN-γ and TNF-α [26]. Trm cells’ role are to produce cytokines for recruitment of effector T cells from the circulation [27]. Through this process, Trm cells mediate long-term maintenance and the potential relapse of vitiligo. Treatments that inhibit this pathway without affecting Trm cell numbers, such as JAK inhibitors, effectively reverse disease, but relapses occur after they are discontinued [28]. We have summarized the most important aspects of pathogenesis of vitiligo in Figure 1.

## 3. Treatment

Vitiligo treatment remains a serious concern and challenge. Topical, systemic and surgical treatments are used for stabilization and repigmentation of vitiligo [29,30]. Treatment modalities are chosen for the individual patient based on the disease severity, disease activity, patient preference and response evaluation. In recent years, efforts have been made to achieve a more comprehensive understanding of vitiligo pathogenesis and to develop novel effective therapies (as targeted therapies) that are able to improve the repigmentation of vitiligo.

### 3.1. JAK Inhibitors

A wide group of JAK inhibitors is currently used to treat a variety of conditions. Depending on the selectivity of each agent, all four JAKs (JAK1, JAK2, JAK3 and TYK2) may be targeted, or, only selected specific kinase may be blocked. In vitiligo treatment, JAK inhibitors inhibit pathogenic T cells targeting melanocytes [31]. However, concerning the final decision of the European Commission: Tofacitinib (Xeljanz), Abrocitinib (Cibinqo), Baricitinib (Olumaint), Upadacitinib (Rinvoq) and Filgotinib (Jyseleca) should only be used if no other suitable alternatives are available in patients 65 years of age and older, patients with a history of atherosclerotic cardiovascular (CV) disease or other CV risk factors, patients who are current or past long-time smokers and patients with malignancy risk factors (e.g., a current or history of malignancy) [32]. Moreover, they should be used with caution in patients with known risk factors for venous thromboembolism [32]. On the other hand, the results of clinical trials in atopic dermatitis had suggested a low risk of serious adverse effects during treatment with JAK inhibitors [33]. Emma Guttman-Yassky et al. suggest that the mentioned discrepancy may be due to significantly younger patients in a group with atopic dermatitis or alopecia areata [33]. Nevertheless, special attention must be noted during treatment with JAK inhibitors, especially in the case of oral agents administered to older individuals.

#### 3.1.1. Topical Ruxolitinib

In two recent phase III clinical trials, the superiority of topical ruxolitinib (JAK1/2 inhibitor) over the placebo in the primary endpoint was shown [34]. Moreover, the results of applying this drug seem to be consistent: 30% of patients have scored a facial Vitiligo Area Scoring Index (fVASI) of ≥75% improvement from the baseline in week 24 [34]. The same was observed in the group previously receiving placebo cream: after a subsequent 28 weeks (24 weeks of vehicle + 28 weeks of ruxolitinib cream), around 30% also scored an improvement of fVASI ≥ 75% [34]. Interestingly, at week 52, around 30% of patients achieved fVASI ≥ 90% and 75% scored fVASI ≥ 50% [34]. No serious adverse effects (SAE) were observed. In the most common AE, application site acne and application site pruritus occurred, both in under 10% of cases [34]. The drug was well tolerated [34]. The results of the phase III trials seem to be consistent with the phase II study [35,36,37]. An important question arises concerning the maintenance of repigmentation after discontinuing the ruxolitinib therapy [37]. The extension of the repigmentation lasted up to 60 months in a phase II study; however, only a small number of patients were included in the study [35,37]. 

In combination therapy (phase II study), the patients receiving Narrow-Band UVB Phototherapy (NB-UVB) had an additional benefit in terms of facial and total body repigmentation when ruxolitinib was added to this method [37,38]. Although the results of the mentioned study seem to be promising, a clinical trial with a higher number of patients is needed to evaluate the efficacy and safety [37]. Multiple studies are underway to determine the optimal drug, method of delivery and use in combination therapy, while NB-UVB is considered to optimize the treatment with topical JAK inhibitors [31].

The perspective of overcoming the limitations of traditional methods of topical drug delivery such as creams, ointments, lotions, gels and other vectors is tempting [39]. Indeed, the nano-drug delivery systems could be the answer [39]. A recent study on ruxolitinib-conjugated gold nanoparticles in the topical treatment of alopecia areata has shown that this method may enhance the efficacy of JAK inhibitors, while minimizing the risk of occurrence of systemic adverse effects [40]. Further studies need to be performed in this area.

The only JAK inhibitor approved by the Food and Drug Administration (FDA) is topical ruxolitinib [37]. Currently, it is registered in the treatment of nonsegmental vitiligo affecting less than 10% of the body surface area (BSA) in adolescents older than 12 years of age and in adults [41].

#### 3.1.2. Oral JAK Inhibitors

Several case reports have been published on the use of oral JAK inhibitors, namely tofacitinib, baricitinib and ruxolitinib [42,43,44,45,46,47,48,49,50]. Despite the initial success of these methods, in some cases, the loss of response and a recurrence of depigmentation were observed [47,49].

An interesting phase IIb clinical trial on ritlecitinib (JAK3 and tyrosine kinase expressed in hepatocellular carcinoma (TEC)) in the treatment of active nonsegmental vitiligo was performed [51]. Both primary and secondary endpoints were met in this study: ritlecitinib 50 mg showed a significantly bigger change from the baseline % in the centrally-read F-VASI than the placebo to week 24 and the proportion of patients who achieved centrally-read F-VASI75 at week 24 was also significantly bigger with ritlecitinib 50 mg (with or without a loading dose) than the placebo [51]. Moreover, accelerated improvement has been noticed after week 28 [51]. Ritlecitinib was well tolerated; however, some patients dropped out of the study, most commonly due to patient withdrawal (8.0%) and secondly due to SAE (5.2%) [37,51]. The 52-week clinical trial of ritlecitinib oral capsules in adults and adolescents with active and stable vitiligo (phase III) is ongoing (NCT05583526).

### 3.2. Prostaglandins and Analogues

Prostaglandins act on numerous cells in human skin, namely keratinocytes, Langerhans cells and melanocytes, which contributes to the increased stimulation of melanocytes and the neural response to stimulation [52]. Additionally, prostaglandins may stimulate the activity and expression of tyrosinase, which is an enzyme limiting the pace of melanin synthesis [52]. As mentioned earlier, oxidative stress has been identified in vitiligo skin. Furthermore, it leads to the reduced synthesis of prostaglandin E2 (PGE2) along with the antagonizing prostaglandin F2 (PGF2α) synthetized in skin [52,53]. PGF2α has been a known marker of oxidative stress and has been recently shown to be elevated (its expression) in vitiligo skin [53].

An original preclinical hypothesis was that the use of PGE may cause repigmentation [52,54]. However, since the discovery of periocular hyperpigmentation caused by PGF2α during therapy of glaucoma, the researchers focused on the possible role of the latter prostaglandin in the clinical studies [52,55,56,57,58,59,60]. In numerous studies, analogues of PGF2α, topical latanoprost and bimatoprost, proved to be an effective additional method of treatment of vitiligo when combined with other methods, NB-UVB, microneedling and momethasone [52,55,56,57,58,59,60]. Currently, a clinical trial comparing topical latanoprost 0.005% ophthalmic solution and 5-fluorouracil in vitiligo treatment is ongoing (NCT05513924).

However, in a recent study, significantly elevated levels of PGF2α in lesional and nonlesional skin of patients with vitiligo were noticed compared to control groups [53]. Due to the contrary result to previous studies, a hypothesis that the periocular hyperpigmentation was caused by the base ingredient of the ointment or solution, not an active prostaglandin, was issued in the literature [53]. Moreover, according to the oxidative stress theory, silymarin (an antioxidant), inhibits melanin synthesis through suppression of PGE2 production by cyclooxygenase-2 (COX-2) [53]. As we have shown earlier, PGE2 is antagonized by PGF2α. Further studies measuring the levels of PGF2α need to be conducted in order to explain the raised inconsistency. 

### 3.3. Afamelanotide

Afamelanotide is the first synthetized α-melanocyte-stimulating hormone (MSH) analogue, which has indeed been shown to be more stable and active than the physiological hormone [61]. This drug targets melanocortin 1 receptor (MC1R) and thereby stimulates the transfer of eumelanin into melanosomes [61]. Interestingly, it has also been shown to induce antioxidant activities, enhance DNA repair processes and modulate inflammation because of the presence of MC1R on the inflammatory cells [52,61]. 

In the biggest study up to date, which was performed on 28 patients with Fitzpatrick scale phototypes III to VI and with a nonsegmental vitiligo involving 15% to 50% of BSA, a superiority of afamelanotide combined with NB-UVB was shown over phototherapy alone (48.64% re-pigmentation versus 33.26% repigmentation (*p* < 0.05)) [62]. The fastest repigmentation has been achieved in the arm of afamelanotide combined with NB-UVB on the face and upper extremities [62]. However, the benefit has been shown only for participants with phototypes IV to VI, not III [62]. The therapy has been well tolerated, the most common AE were erythema and nausea [62].

Another phase II study has revealed the supremacy of afamelanotide in combination with NB-UVB in comparison with NB-UVB alone [63]. Currently, another clinical trial, which targets in order to assess the change in pigmentation and safety of afamelanotide in patients with vitiligo on the face is ongoing (NCT05210582). Importantly, afamelanotide may be considered as a promising addition to the phototherapy; however, only the latter method itself is able to induce melanoblast differentiation [52]. Therefore, obtaining more data on the safety of the treatment, long-term efficacy on the larger number of patients and the effect on I and II Fitzpatrick phototypes is essential to consider implementing this therapy on a broader scale [52].

### 3.4. Other Potential Treatments

#### 3.4.1. Cell-Based Therapies

Various cell-based therapies used in the treatment of vitiligo have been recently summarized in a published review [64]. Out of melanocyte transplantation, melanocyte–keratinocyte cell transplantation (MKCT), ReCell, non-cultured epidermal cell grafting and combination therapy, the MKCT seems to be the most effective method [64]. In most cases, performing MKCT results in at least 90% of repigmentation, which is superior to other methods [64,65]. Despite being safe, well tolerated and bringing excellent results in terms of percentages of repigmentation, surgical techniques have several limitations [64,65]. First, according to the algorithm of vitiligo treatment, surgical treatment should be considered in the case of stable disease lasting longer than one year and affecting less than 5–10% of BSA [65]. Importantly, when treating segmental vitiligo, the decision to implement cell-based therapies may be earlier depending on the course of the vitiligo [65]. Further limitations are the cost, the need for the practitioner to know the procedure and the necessity to have specialist equipment that is essential to perform these surgeries [64]. Lastly, several states exclude patients from cell-based therapies, such as koebnerization, including hypertrophic scarring, keloids and patients with susceptibility to poor wound healing [64]. Therefore, in case of good patient selection, cell-based techniques may be an excellent way to treat vitiligo.

#### 3.4.2. Wnt/β-Catenin-Signaling Agonists

Wnt/β-catenin-signaling is downregulated in patients with vitiligo and its upregulation may contribute to controlling the immune response and thereby protect melanocytes from oxidative stress, inhibit CD8+ cytotoxic T lymphocytes and activate Tregs [66]. Several activators and upregulators of this signaling pathway such as simvastatin or lithium chloride may have an additional role in vitiligo treatment [66]. However, up to date only simvastatin was researched in phase II clinical trials and in most studies no correlation between oral uptake of this agent and repigmentation has been identified [67,68]. Despite simvastatin’s possible role in improving the control of repigmentation, it should be indicated in patients in order to lower cholesterol and triglyceride levels, not in the treatment of vitiligo per se.

## 4. Future Directions

Many possible concepts for vitiligo treatment are continuously developed while our understanding of vitiligo pathogenesis is increasing [69,70]. Investigating the role of genetics, immune system dysfunction, oxidative stress, and neural factors should be included in these considerations. Increased knowledge in these areas could lead to the development of more targeted and effective treatments. The use of IL-15 inhibitors seems tempting because as we have shown earlier that this cytokine has an important role in the promotion of the Trm lymphocytes subset [71]. Blocking IL-15 or its receptor may be effective and is currently studied in an ongoing phase IIa clinical trial (NCT04338581). CD8+ T lymphocyte toxicity is also an important component of vitiligo pathogenesis, therefore therapies blocking IFN-γ, the IFN-γ receptor, the ligands CXCL10 and CXCL9 and the receptor CXCR3 may also be worth exploring and testing in clinical studies [70]. The further argument supporting this thesis is the fact that some JAK inhibitors, which act on the same cytokine–receptor JAK/STAT pathways have proven to be efficient in phase III randomized clinical trials. The next factor also contributing to the cytotoxic microenvironment in vitiligo is mentioned before inducible HSP70 (HSP70i) DNA [69]. Therefore, blocking HSP70i may be a good strategy to downregulate the Th-1 lymphocyte phenotype of active vitiligo lesions. On the other hand, several cases of new-onset vitiligo after administration of drugs inhibiting Th-1 cytotoxicity have been reported. Most frequently, the drugs responsible were TNF-alpha inhibitors (adalimumab, inxliximab and certolizumab), anti-IL-12/IL-23 monoclonal antibody (ustekinumab) and anti-IL17a (secukinumab) (in just one case) [72,73,74]. The mentioned data remind us of the possible dangers of new agents and prompt the accurate and frequent examination of patients receiving new drugs. High mobility group box protein B1 (HMGB1) may be another important target for the treatment of vitiligo [75]. HMGB1 is also one of the DAMPs and their levels are increased in the sera of vitiligo patients. DAMPs induce melanocyte apoptosis and may be involved in recognizing autoantigens and regulating the immune response. Investigating the effects of HMGB1 on melanocytes may be valuable for enhancing our understanding of vitiligo and developing new therapeutics. Another study on vitiligo treatment demonstrated the usefulness of adipose-derived stem cells (ADSCs). Their regenerative function and immunomodulatory properties make them therapeutically potent. Unfortunately, locally administered stem cells might not be sufficient to reverse the pathological mechanisms and furthermore, the damaged tissue remodeling process requires further studies [76]. Tailoring the treatment to an individual’s specific genetic and immune profile could enhance treatment outcomes and reduce side effects. Vitiligo may have a significant impact on a person’s psychological well-being and quality of life. Future directions in the field may involve developing comprehensive support systems and interventions to address the emotional and social aspects of living with this disease. We should not forget about increasing public awareness about vitiligo which could help reduce the stigma associated with the condition and promote acceptance and understanding. Advocacy efforts may drive research funding and support initiatives for improved access to treatment. Importantly, while these directions show promise, research and development both take time. Several years of studies for these potential advancements to become widely available are needed in order to have a significant impact on the management and treatment of vitiligo. Several years of studies are needed for these potential advancements to become widely available and have a significant impact on the management and treatment of vitiligo.

**Figure 2:**
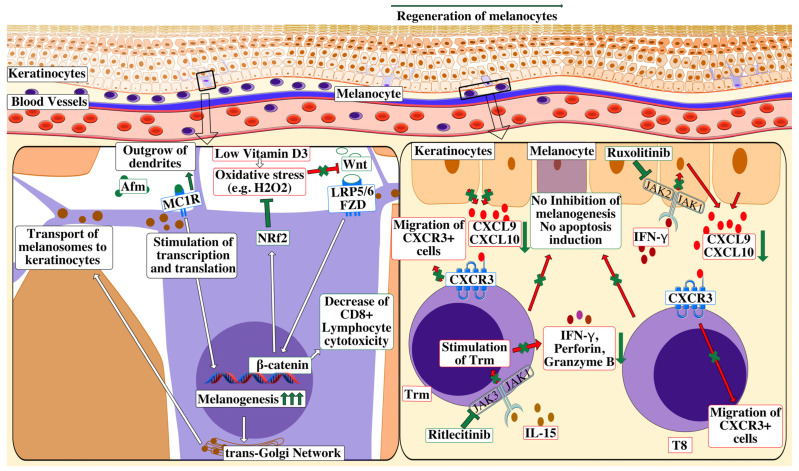
Vitiligo: treatment targets of novel therapies. Pathogenesis of vitiligo and treatment targets of selected therapies: T8 expression of IFN-γ in vitiligo lesions activates the JAK/STAT pathway after binding to the IFN-γ receptor, thus facilitating the release of CXCL9/10 from the keratinocytes. The binding of CXCL9/10 to CXCR3 increases CXCR3+ T cell recruitment; along with the depigmentation, more T-cells appear at the site of inflammation. The pathogenic function of IL-15-dependent Trm is to produce IFN-γ, perforin and granzyme B, namely cytokines with cytotoxic properties. Along with T8, the Trm contributes to inhibition of melanogenesis and induces the apoptosis of melanocytes. Ruxolitinib (an inhibitor of JAK1/JAK2) stops the transmission of the IFN-γ/CXCL9/10 and CXCR3 axis. Furthermore, ritlecitinib as an inhibitor of JAK3, which may contribute to the decrease in Trm cell activation and their release of IFN- γ, perforin and granzyme B. The Wnt/β-catenin pathway has been shown to be down regulated in vitiligo. Wnt acts through the activation of FZD and LRP5/6, thereby activating B-catenin epigenetic properties. OS inhibits the Wnt/β-catenin pathway activation. Low vitamin D levels fail to reverse the inhibitory effects of OS. In the case of an agent stimulating Wnt/β-catenin (e.g., statins), it has been theorized that the beneficial function of NRf2 lies in inhibiting OS and indeed a decrease in T8 cytotoxicity may be noticed. Lastly, afm, the analogue of α-MSH, acts through activation of MC1R. Then, the transcription, translation, proper folding, and transport into melanosomes of tyrosinase and tyrosinase-related proteins are stimulated, which propels melanogenesis. Next, the outgrowth of dendrites is promoted and melanosomes are distributed to nearby keratinocytes. Lastly, MC1R signaling promotes DNA repair and synthesis of antioxidant enzymes, reducing the OS. Abbreviations: T8—cytotoxic CD8+ lymphocyte T, Trm—resident memory T cells, JAK—Janus kinase, STAT—signal transducer and activator of transcription, CXC9/10—chemokine (C-X-C motif) ligand 9/10, CXCR3—chemokine (C-X-C motif) receptor 3, FZD—Frizzled receptor, LRP5/6—lipoprotein receptor-related protein 5/6, IFN-γ—interferon-gamma, NRf2—nuclear factor erythroid 2-related factor 2, OS—oxidative stress, afm—afamelanotide, α-MSH—α-melanocyte-stimulating hormone and MC1R—melanocortin-1 receptor.

Novel and future therapies are summarized in Figure 2.

## 5. Conclusions

The treatment and management of vitiligo has remained an intractable challenge for both scientists and dermatologists. Current models of treatment are often nonspecific. Recently, a better understanding of the pathophysiological processes of vitiligo led to the advent of more targeted, effective and safe treatments for patients who suffer from this disease. IFN-γ-induced chemokines activate the JAK-STAT signaling pathway and promote the migration of further autoreactive T cells (CD8 +) which exacerbates inflammation via the positive feedback mechanism. This IFN-γ-chemokine signaling axis is responsible for progression and maintenance of vitiligo. Therapies that disrupt the pathway targeting IFN-γ, the IFN-γ receptor, the downstream signal JAK-STAT pathway, CXCL10 and its receptor CXCR3 have been some of the most promising in vitiligo management and may become the first to be approved. Unfortunately, during the first year after stopping treatment, a relapse of the depigmentation is observed. It occurs mainly at the same areas that were previously involved and is mediated by autoreactive Trm cells. Their role is to produce cytokines for recruitment of effector T cells from the circulation. Through this process, Trm cells mediate long term maintenance and the potential relapse of vitiligo. Treatments that inhibit this pathway without affecting the Trm cell number, such as JAK inhibitors, effectively reverse disease but relapses occur after they are discontinued. Targeting their maintenance in the skin through IL-15 or other approaches may prove to be a more durable treatment strategy. Future therapeutical methods may reverse disease by affecting skin homeostasis rather than simply inhibiting inflammation. The stimulation of melanocyte stem cell regeneration, the activity and expression of tyrosinase and the synthesis of α- MSH analogue may replace phototherapy treatments and synergize with immune therapies to produce more effective treatment strategies. Many unanswered questions regarding vitiligo initiation and progression still exist and this provides new opportunities for additional discoveries.

## Data Availability

No new data were created or analyzed in this study. Data sharing is not applicable to this article.
